# Supplementary material for: The contribution of genetic determinants of blood gene expression and splicing to molecular phenotypes and health outcomes
Source: Nat Genet. 2025 Mar 4;57(3):616–25. doi: 10.1038/s41588-025-02096-3 (PMC11906350; doi:10.1038/s41588-025-02096-3)
Supplement: Supplementary file 1 — Supplementary Figs. 1–10 and Note. [file 41588_2025_2096_MOESM1_ESM.pdf]

nature genetics

Supplementary information https://doi.org/10.1038/s41588-025-02096-3

The contribution of genetic determinants of blood gene expression and splicing to molecular phenotypes and health outcomes

In the format provided by the authors and unedited

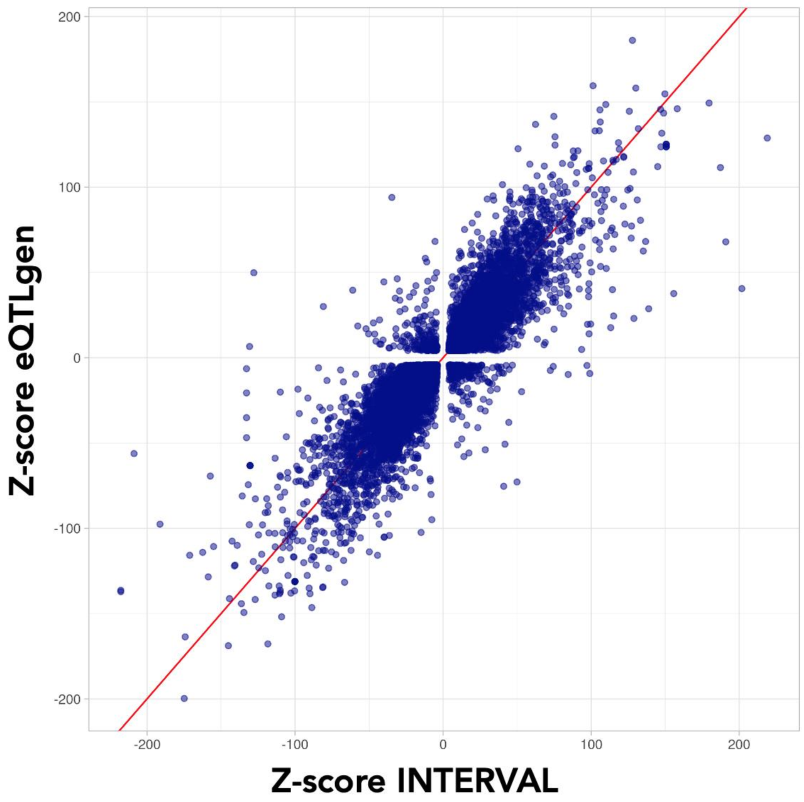

Supplementary Figure 1. Correlation of *cis*-eQTL Z-scores between INTERVAL and eQTLGen.

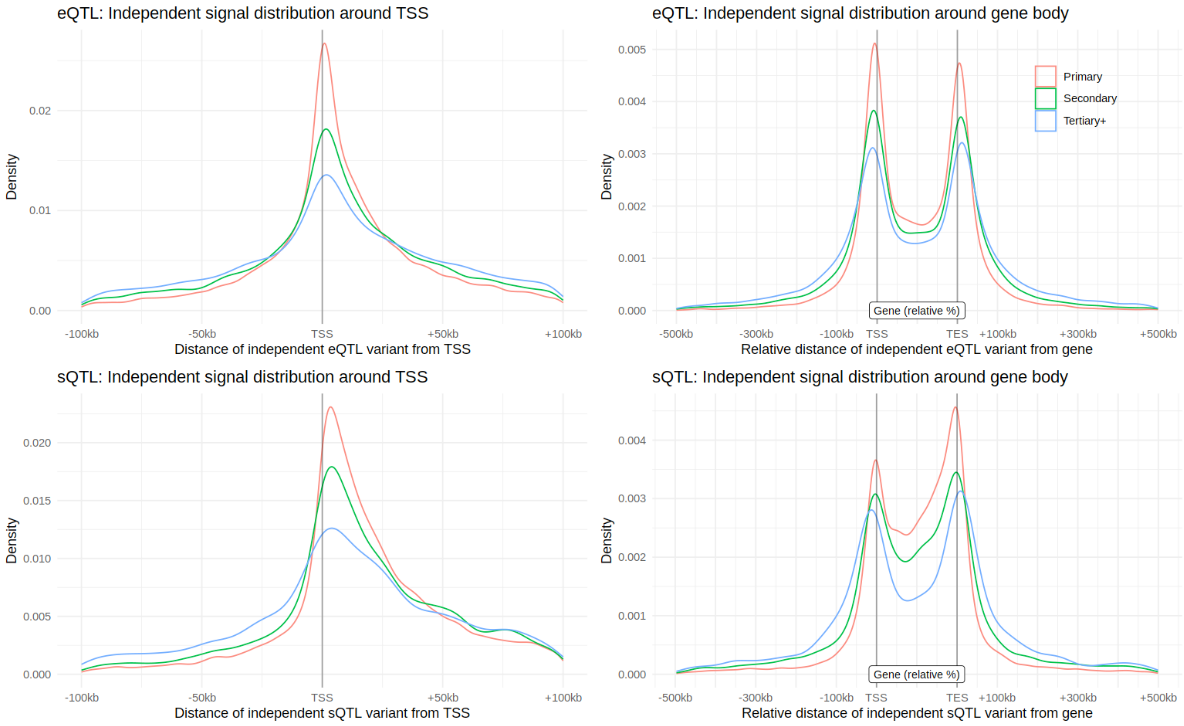

Supplementary Figure 2. *Cis*-eQTL and -sQTL independent signal distribution around TSS and gene boundaries.

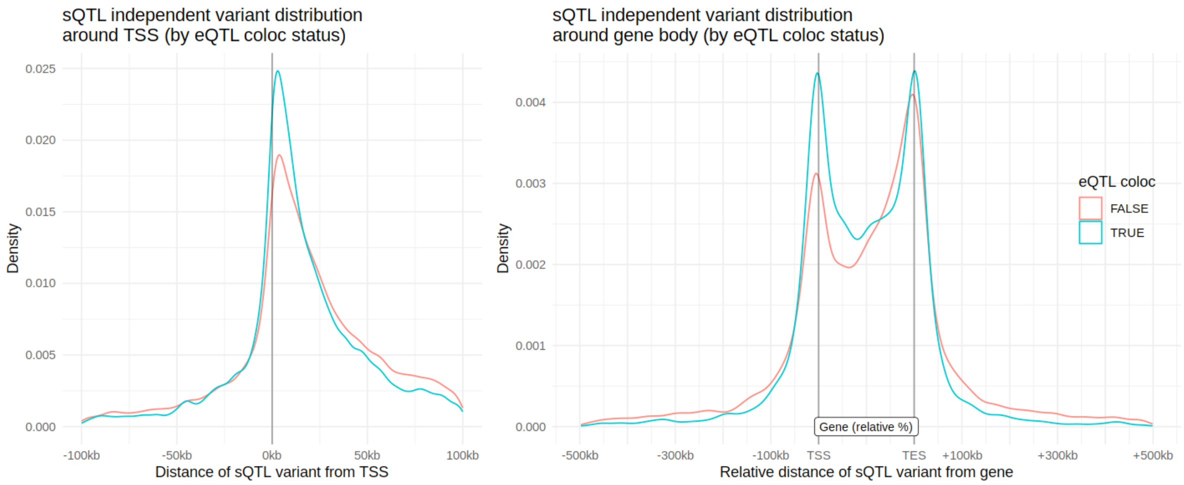

Supplementary Figure 3. *Cis*-eQTL and -sQTL colocalization analysis and lead SNP distribution around the TSS.

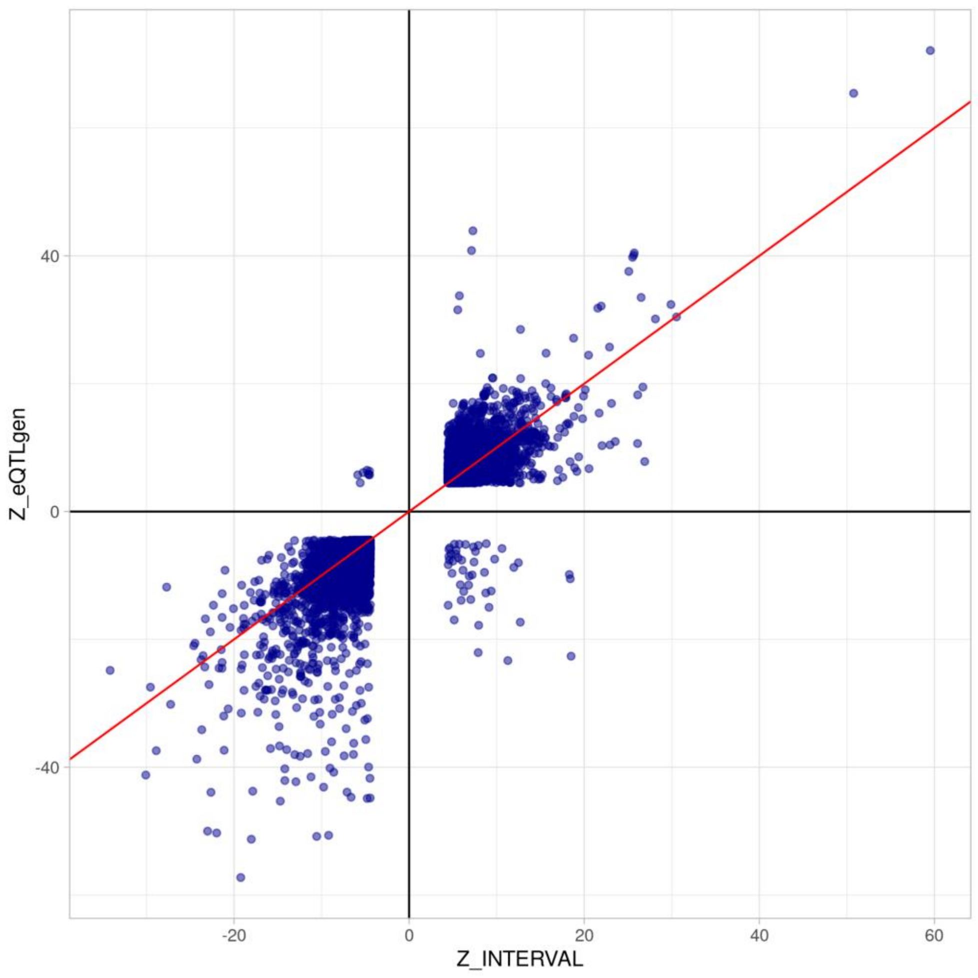

Supplementary Figure 4. Correlation of *trans*-eQTL Z-scores between INTERVAL and eQTLGen. Z-scores were plotted for SNP–gene pairs, for which there is a significant *trans*-association in eQTLGen, and that are also available in our all-vs-all *trans*-eQTL results.

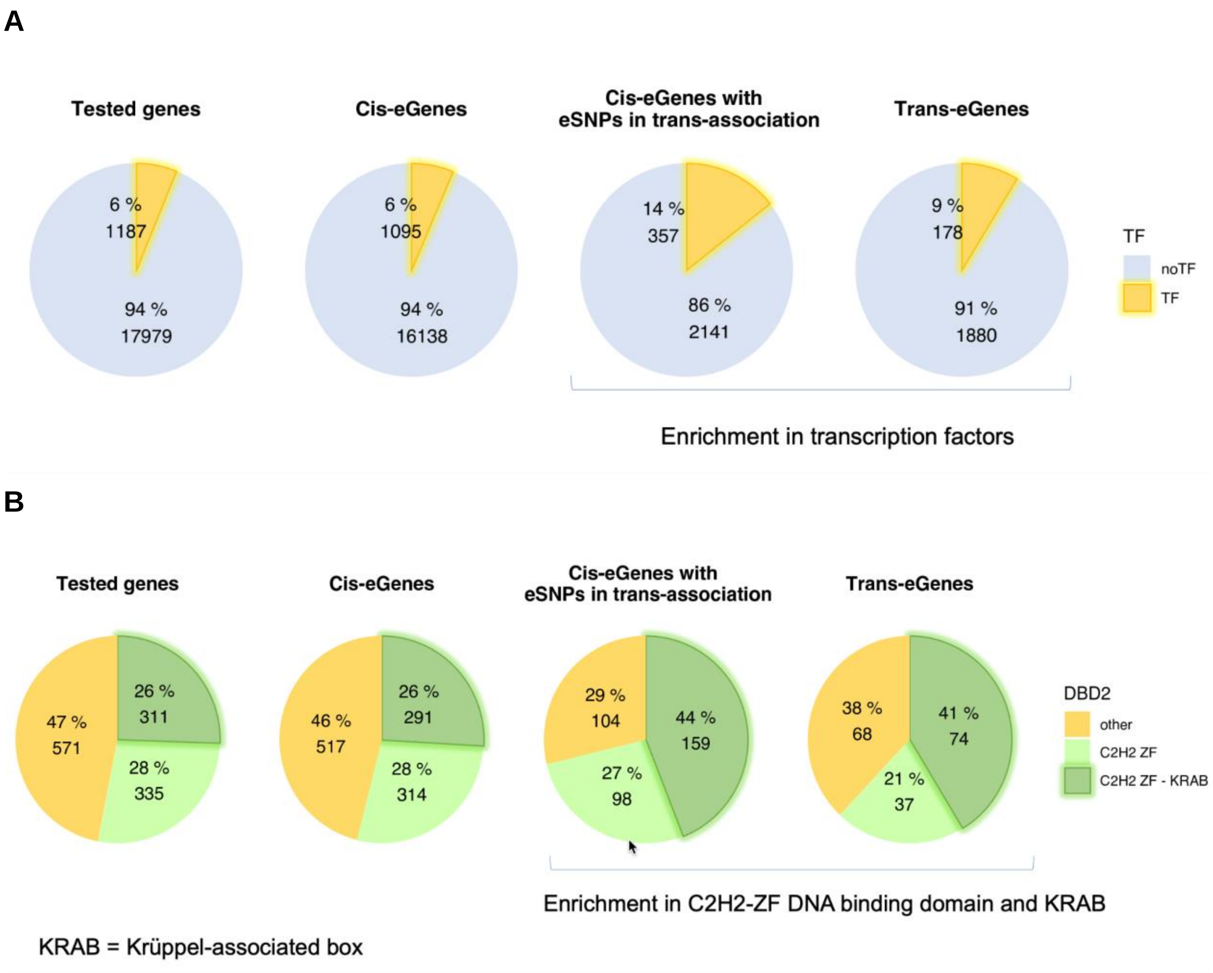

Supplementary Figure 5. Transcription factor annotation in *cis*- and *trans*-QTL results. A. Proportion of transcription factors in *cis*- and *trans*-eGenes; B. Subcategories of transcription factors in *cis*- and *trans*-eGenes.

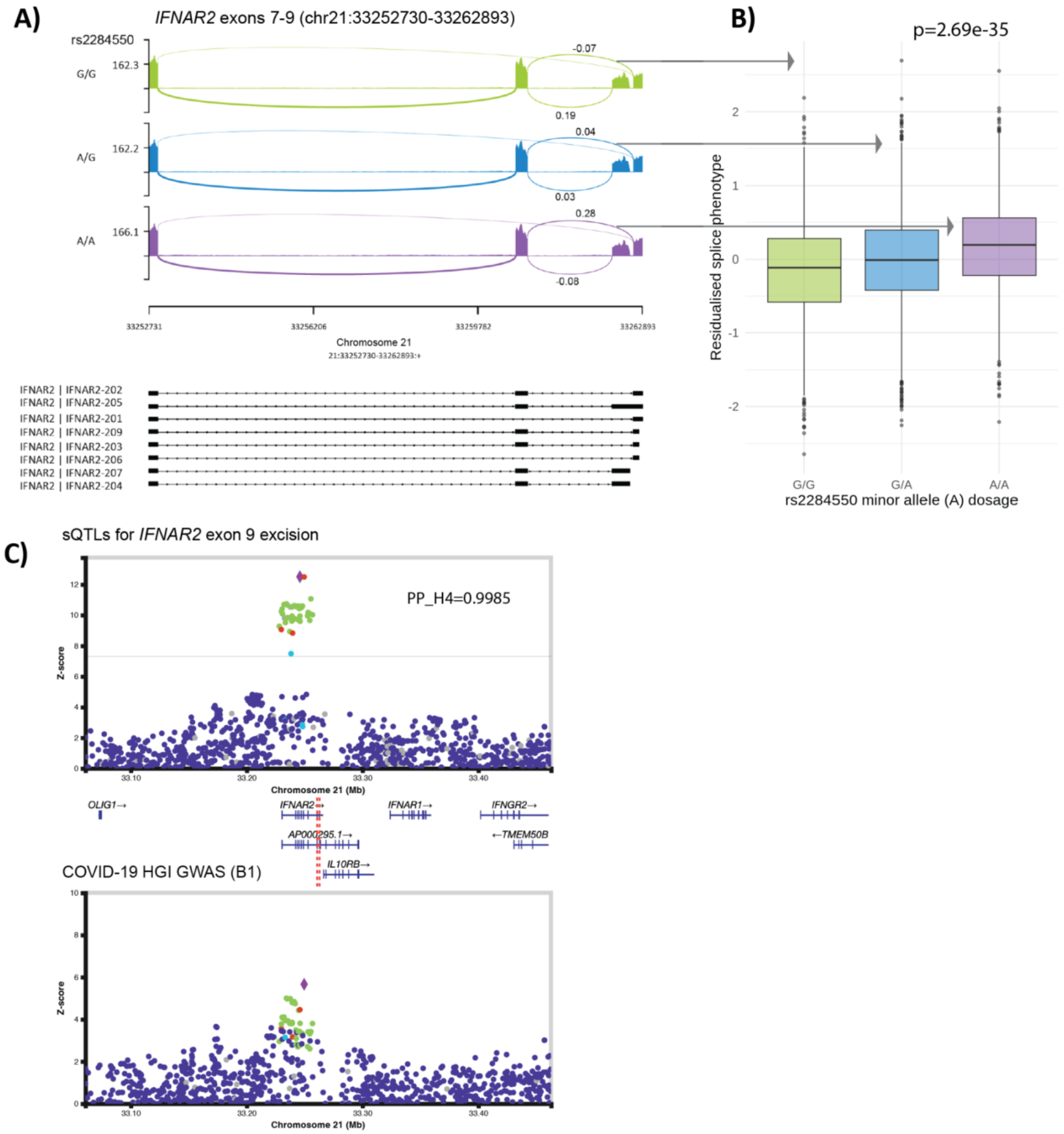

Supplementary Figure 6. Transcriptional mechanisms underlying the *IFNAR2* locus associated with COVID-19 susceptibility and severity. A. Sashimi plot demonstrating the cluster of splice events surrounding exons 7-9 of *IFNAR2*. These represent those with highest posterior probability of colocalization with the COVID19-HGI GWAS, annotated with median splice event phenotype value per genotype. This is grouped by the minor allele count for the lead SNP of the splice event with the highest posterior probability of colocalization. B. Boxplot for the lead SNP for the transcript splicing phenotype excising part of the last exon. C. QTL plot of the variants at this signal for both the transcript splicing phenotype and the COVID-HGI GWAS.

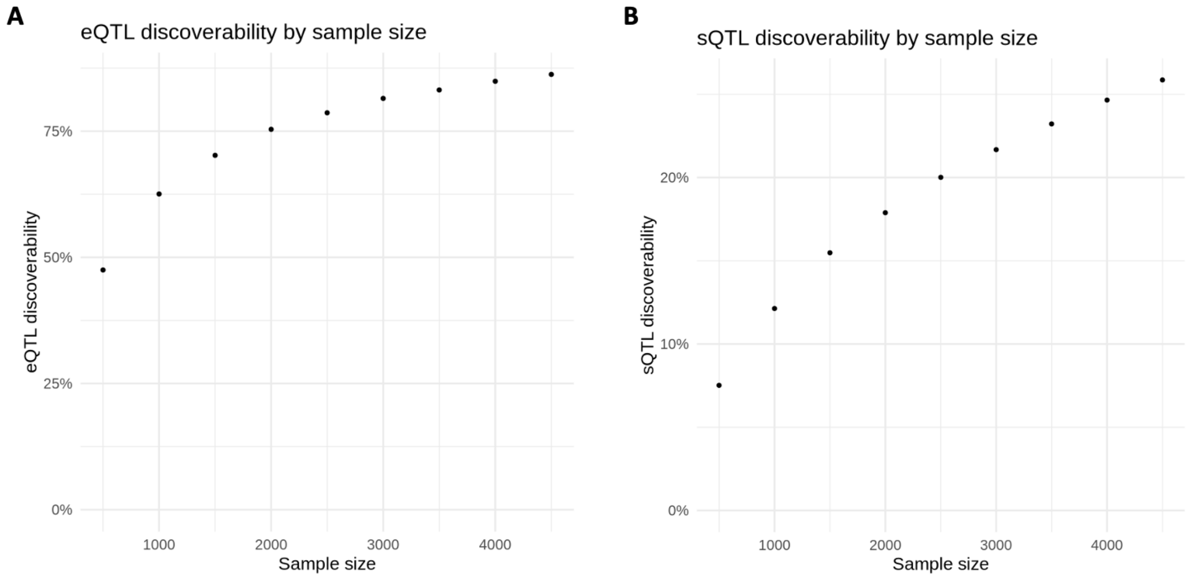

Supplementary Figure 7. *cis*-eQTL and *cis*-sQTL discovery rate as a function of sample size. Down-sampling analysis was performed for decreasing subsets of individuals from the full cohort. The percentage of genes with a significant *cis*-eQTL (A) and splice events with a *cis*-sQTL (B) was subsequently calculated.

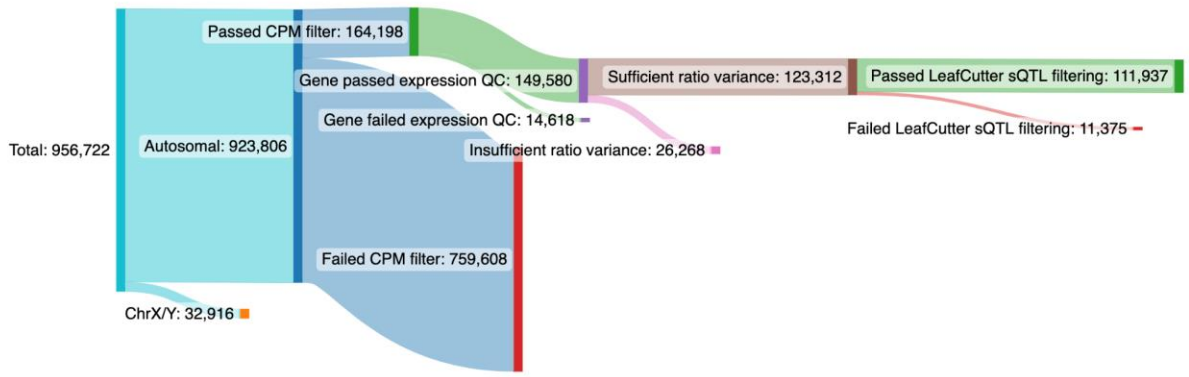

Supplementary Figure 8. Summary of splice event QC.

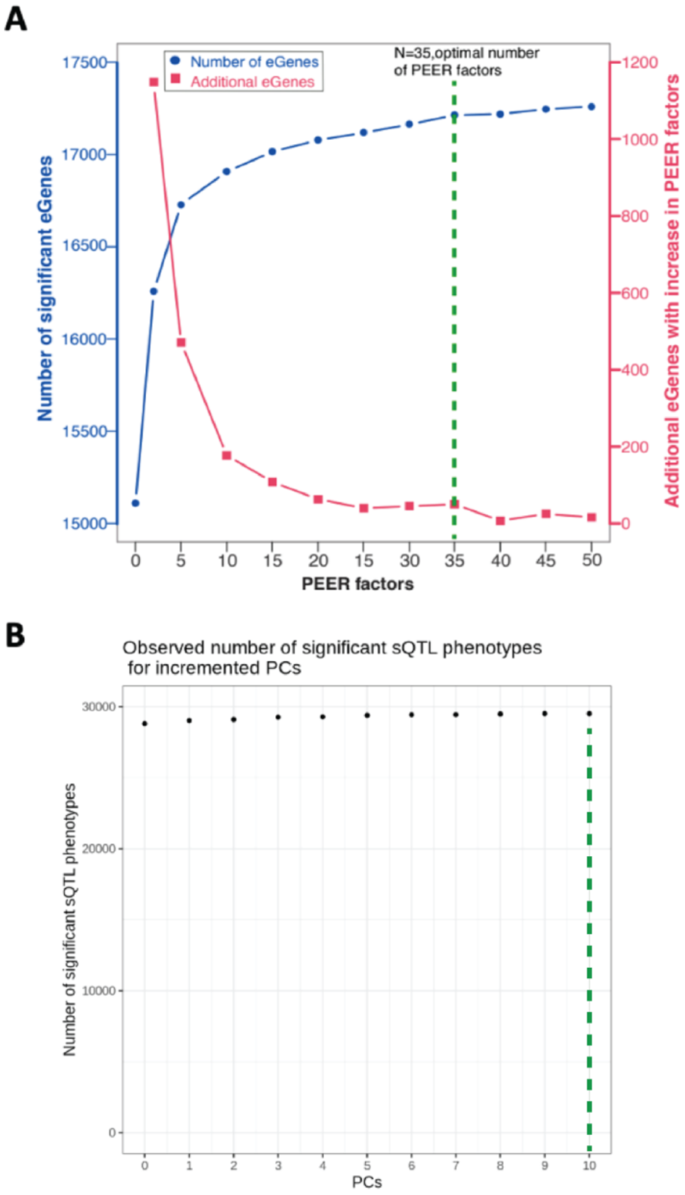

Supplementary Figure 9. Identification of the optimal number of PEER factors (latent variation estimated from gene expression data) and PCs to be included as covariates in the eQTL and sQTL analyses. A. PEER factors were chosen based on the maximum number of *cis*-eGenes discovered (Y-axis, left) and the gain in *cis*-eGenes with incremental increase in PEER factors (Y-axis, right). B. *cis*-sQTL discovery with inclusion of increasing numbers of principal components in the model.

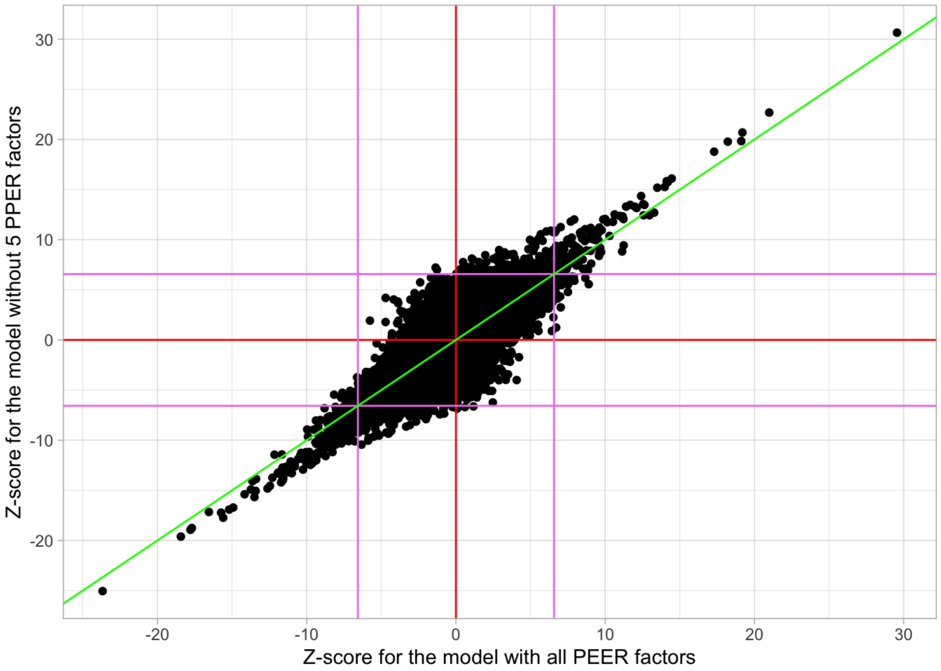

Supplementary Figure 10. Comparison of *trans*-eQTL Z-scores for ten SNPs significantly associated with a PEER factor, before and after removing the five PEER factors associated with *cis*-eSNPs from model covariates. Ten *cis*-eSNPs were found significantly associated with 5 PEER factors (Bonferroni multiple testing correction: $p < 0.05$ / 53,457 *cis*-eSNPs / 35 PEER factors = 2.7×10^{-8}): rs7310615, rs17630235, rs3809272 (chromosome 12) associated with PEER factor 10; rs74505413, rs144476978, rs4795085, rs75715226 (chromosome 17) associated with PEER factor 12; rs11168070 (chromosome 5) associated with PEER factor 26; rs72878029 (chromosome 11) associated with PEER factor 13; and rs9264670 (chromosome 6) associated with PEER factor 7. The Pearson correlation of Z-scores for *trans*-eQTL involving these ten SNPs before and after removing these PEER factors from the model is 0.80. Pink lines are drawn at the significance threshold of $p=5\times10^{-11}$ for the identification of *trans*-eQTLs.

Supplementary Note 1

To provide experimental validation, we overlapped our *trans*-sQTL associations with the RBP binding and alternative splicing upon knockdown analyses in K562 and HepG2 cells performed by the ENCORE project (Van Nostrand, E.L. et al. A large-scale binding and functional map of human RNA-binding proteins. *Nature* 583, 711-719 (2020)). As *QKI* was the only gene also tested by the authors, we limited our analysis to this gene (Supplementary Table 12). We found that 12/18 sGenes were reported as eCLIP targets of QKI, and 8/18 of these reported significant alternative splicing differences in ENCORE. Further, we observed that genes that were *trans*-sQTL sGenes in our analysis had more significant eCLIP binding (p=0.021, one-sided Wilcoxon test) and a significantly greater absolute ΔPSI upon knockdown (p=0.021) compared to those that were not.
