# Supplementary material for: The contribution of genetic determinants of blood gene expression and splicing to molecular phenotypes and health outcomes
Source: Nat Genet. 2025 Mar 4;57(3):616–25. doi: 10.1038/s41588-025-02096-3 (PMC11906350; doi:10.1038/s41588-025-02096-3)
Supplement: Supplementary file 2 — Reporting Summary [file 41588_2025_2096_MOESM2_ESM.pdf]

nature portfolio

Corresponding author(s): Dirk S. Paul

Last updated by author(s): Nov 14, 2024

Reporting Summary

Nature Portfolio wishes to improve the reproducibility of the work that we publish. This form provides structure for consistency and transparency in reporting. For further information on Nature Portfolio policies, see our Editorial Policies and the Editorial Policy Checklist.

Statistics

For all statistical analyses, confirm that the following items are present in the figure legend, table legend, main text, or Methods section.

n/a	Confirmed	
☐	☒	The exact sample size (*n*) for each experimental group/condition, given as a discrete number and unit of measurement
☐	☒	A statement on whether measurements were taken from distinct samples or whether the same sample was measured repeatedly
☐	☒	The statistical test(s) used AND whether they are one- or two-sided<br>*Only common tests should be described solely by name; describe more complex techniques in the Methods section.*
☐	☒	A description of all covariates tested
☐	☒	A description of any assumptions or corrections, such as tests of normality and adjustment for multiple comparisons
☐	☒	A full description of the statistical parameters including central tendency (e.g. means) or other basic estimates (e.g. regression coefficient) AND variation (e.g. standard deviation) or associated estimates of uncertainty (e.g. confidence intervals)
☐	☒	For null hypothesis testing, the test statistic (e.g. *F*, *t*, *r*) with confidence intervals, effect sizes, degrees of freedom and *P* value noted<br>*Give P values as exact values whenever suitable.*
☐	☒	For Bayesian analysis, information on the choice of priors and Markov chain Monte Carlo settings
☒	☐	For hierarchical and complex designs, identification of the appropriate level for tests and full reporting of outcomes
☐	☒	Estimates of effect sizes (e.g. Cohen's *d*, Pearson's *r*), indicating how they were calculated

Our web collection on statistics for biologists contains articles on many of the points above.

Software and code

Policy information about availability of computer code

Data collection	Genotyping and RNA-seq data collection was performed using the standard vendor bioinformatic pipeline for the Affymetrix Axiom UK Biobank genotyping array and the NovaSeq 6000 respectively. Details for the collection of previously generated protein and metabolite data are listed in Supplementary Table 29, and associated manuscripts. Covariate and population meta-data were previously collected as described in 10.1016/S0140-6736(17)31928-1.
Data analysis	Software: CrossMap v0.3.4, FastQC v0.11.8, featureCounts v2.0.0, GCTA-COJO v1.94.0beta (January 2022), Leafcutter v0.2.9, PLINK v1.9, PLINK v2.00a2-32-bit, QTLtools MBV v1.2, R v4.0.3, regtools v0.5.2, SHAPEIT3, STAR v2.7.3a, tensorQTL v1.0.6.<br>R packages: biomaRt v2.46.3, coloc v5.1.0.1, edgeR v3.24.3, GenABEL v1.8-0, medflex v0.6-7, peer v.1.0.<br>Interactive QTL web-portal: Bootstrap v3.4.1, DataTables v1.13.3, jquery v3.5.1, LocusZoomJS v0.13.4, MariaDB v10.2.38, PHP v7.2.34, plotly v2.9.0.<br>Further information about the software and R packages used for data analyses are provided in the Methods section of the manuscript. The main code for analyses is available at https://github.com/INTERVAL-RNAseq/manuscript-scripts.

For manuscripts utilizing custom algorithms or software that are central to the research but not yet described in published literature, software must be made available to editors and reviewers. We strongly encourage code deposition in a community repository (e.g. GitHub). See the Nature Portfolio guidelines for submitting code & software for further information.

Data

Policy information about availability of data

All manuscripts must include a data availability statement. This statement should provide the following information, where applicable:

- Accession codes, unique identifiers, or web links for publicly available datasets
- A description of any restrictions on data availability
- For clinical datasets or third party data, please ensure that the statement adheres to our policy

The INTERVAL study data used in this paper are available to bona fide researchers from ceu-dataaccess@medschl.cam.ac.uk. The data access policy for the data is available at http://www.donorhealth-btru.nihr.ac.uk/project/bioresource. The newly generated RNA-sequencing data (n=4,732 INTERVAL participants) have been deposited at the European Genome-phenome Archive (EGA) under the accession number EGAD00001008015. The results from the genetic association, colocalization and mediation analyses are available in the Supplementary Tables, and online at https://IntervalRNA.org.uk. The full summary statistics are also made available on the above web portal, as well as mirrored on Zenodo (10354433). For external and previously computed summary statistics for proteins and metabolites, these accession details are listed in Supplementary Table 29 and associated manuscripts. GTEX v8 summary statistics were downloaded from the online repository (https://gtexportal.org/), and eQTLGen (Phase 1) from their repository (https://www.eqtlgen.org/). The GRCh38 reference genome was sourced from NCBI (https://www.ncbi.nlm.nih.gov/datasets/genome/GCF_000001405.26/) and annotations from the Ensembl V99 GTF (https://www.ensembl.org/). For enrichment analyses, we used the public databases Gene Ontology (https://geneontology.org/) and the Human Transcription Factors database (https://humantfs.ccbr.utoronto.ca/).

Research involving human participants, their data, or biological material

Policy information about studies with human participants or human data. See also policy information about sex, gender (identity/presentation), and sexual orientation and race, ethnicity and racism.

Reporting on sex and gender	Study participants were recruited as part of the INTERVAL study. For the analysis of the RNA-sequencing data, there were 2,105 female and 2,627 male participants.
Reporting on race, ethnicity, or other socially relevant groupings	Participants from the INTERVAL study were recruited across England, UK. After quality control of the genotype data, remaining participants were from European ancestry only. No prior ethnicity information was used for filtering participants.
Population characteristics	Participants were generally in good health as blood donation criteria excluded individuals with a history of major diseases (e.g., myocardial infarction, stroke, cancer, HIV, and hepatitis B or C) and who had a recent illness or infection. Participant age ranged from 20-79, with a median of 58. Additional population demographic characteristics are described in Supplementary Table 27.
Recruitment	Between 2012 and 2014, blood donors aged 18 years and older were recruited at 25 centers of England's National Health Service Blood and Transplant (NHSBT).
Ethics oversight	All participants gave informed consent before joining the study and the National Research Ethics Service approved this study (11/EE/0538).

Note that full information on the approval of the study protocol must also be provided in the manuscript.

Field-specific reporting

Please select the one below that is the best fit for your research. If you are not sure, read the appropriate sections before making your selection.

☒ Life sciences ☐ Behavioural & social sciences ☐ Ecological, evolutionary & environmental sciences

For a reference copy of the document with all sections, see nature.com/documents/nr-reporting-summary-flat.pdf

Life sciences study design

All studies must disclose on these points even when the disclosure is negative.

Sample size	Raw RNA-sequencing data included 4,778 individuals. After quality control, the RNA-sequencing data included 4,732 individuals. No methodology was used to predetermine sample size, though this study is more than 7 times larger than previous similar studies. Sample sizes of external datasets that were integrated in the different analyses are described in the Supplementary Table 29.
Data exclusions	We filtered samples of poor quality by removing samples with a read depth below 10 million uniquely mapped reads. A relatedness matrix was obtained using the PLINK v1.9 -make-rel 'square' command on pruned genotype data, and a cut-off threshold of 0.1 was used to define related individuals. For each pair of related individuals, one individual was arbitrarily removed. After quality control, a total of N=46 samples were removed.
Replication	We did not replicate our eQTL and sQTL mapping in a separate dataset but validated our results by performing a comparison with results obtained from external studies (i.e., eQTLGen Consortium and GTEx Consortium studies). We observed Pearson's r=0.9 for cis-eQTL z-score overlap with eQTLGen, and Pearson's r=0.9 for trans-eQTLs. For cis-sQTLs, 89.0% of the 2,677 GTEX whole blood sGenes we also tested were

	also found as sGenes in our analysis. For trans-sQTLs, there were only 2 results in GTEX whole blood, both of which we replicated.
Randomization	Participants were randomly selected from the INTERVAL study, a cohort comprising presumably healthy blood donors, irrespective to covariate status.
Blinding	Blinding was not relevant to this study as there was no case-control status.

Reporting for specific materials, systems and methods

We require information from authors about some types of materials, experimental systems and methods used in many studies. Here, indicate whether each material, system or method listed is relevant to your study. If you are not sure if a list item applies to your research, read the appropriate section before selecting a response.

Materials & experimental systems

n/a	Involved in the study
☒	☐ Antibodies
☒	☐ Eukaryotic cell lines
☒	☐ Palaeontology and archaeology
☒	☐ Animals and other organisms
☒	☐ Clinical data
☒	☐ Dual use research of concern
☒	☐ Plants

Methods

n/a	Involved in the study
☒	☐ ChIP-seq
☒	☐ Flow cytometry
☒	☐ MRI-based neuroimaging

Plants

Seed stocks	N/A
Novel plant genotypes	N/A
Authentication	N/A
